# Evolution, Expression and Functional Analysis of CXCR3 in Neuronal and Cardiovascular Diseases: A Narrative Review

**DOI:** 10.3389/fcell.2022.882017

**Published:** 2022-06-20

**Authors:** Devi Satarkar, Chinmoy Patra

**Affiliations:** ^1^ Department of Developmental Biology, Agharkar Research Institute, Pune, India; ^2^ SP Phule University, Pune, India

**Keywords:** CXCR3, disease, cardiovascular, nervous system, zebrafish, chemokines

## Abstract

Chemokines form a sophisticated communication network wherein they maneuver the spatiotemporal migration of immune cells across a system. These chemical messengers are recognized by chemokine receptors, which can trigger a cascade of reactions upon binding to its respective ligand. CXC chemokine receptor 3 (CXCR3) is a transmembrane G protein-coupled receptor, which can selectively bind to CXCL9, CXCL10, and CXCL11. CXCR3 is predominantly expressed on immune cells, including activated T lymphocytes and natural killer cells. It thus plays a crucial role in immunological processes like homing of effector cells to infection sites and for pathogen clearance. Additionally, it is expressed on several cell types of the central nervous system and cardiovascular system, due to which it has been implicated in several central nervous system disorders, including Alzheimer’s disease, multiple sclerosis, dengue viral disease, and glioblastoma, as well as cardiovascular diseases like atherosclerosis, Chronic Chagas cardiomyopathy, and hypertension. This review provides a narrative description of the evolution, structure, function, and expression of CXCR3 and its corresponding ligands in mammals and zebrafish and the association of CXCR3 receptors with cardiovascular and neuronal disorders. Unraveling the mechanisms underlying the connection of CXCR3 and disease could help researchers investigate the potential of CXCR3 as a biomarker for early diagnosis and as a therapeutic target for pharmacological intervention, along with developing robust zebrafish disease models.

## Introduction

With billions of circulating cells, the immune system relies on a very complicated yet efficient communication system to guide these cells along their complex pathways and choreograph interactions between them. Chemokines are part of this elaborate communication network and work through the body in the form of “messages,” which are decoded by specific cell-surface receptors. In the presence of a chemokine ligand, a specific receptor activates particular functional responses in the cells that express them. Chemokines are best-known for their property of prompting directional migration of cells, which plays a vital role in development and organ homeostasis, immune cell homing, angiogenesis, and in disease conditions like inflammation, autoimmunity, and cancer (Allen et al., 2007).

Depending on the kind of responses they mount in cells, there are two basic types of chemokines, inflammatory and homeostatic, though most chemokines play a dual role (Allen et al., 2007). Chemokines have been divided into four subfamilies based on cysteine residues at the N-terminal; CC chemokines have two adjacent cysteines, while CXC chemokines have an amino acid between them. Two other subfamilies are C and CX3C chemokines, also called lymphotactin and fractalkine, respectively (Zlotnik and Yoshie, 2000). These chemokines have their own set of complementary receptor families. CXC chemokines, also known as CXC ligands (CXCLs) that interact with CXC chemokine receptors (CXCRs), belong to a G protein-coupled seven-transmembrane receptor family (Mantovani, 1999). CXCR family comprises seven receptors, CXCR1-7, and there is tremendous promiscuity in chemokine binding; one receptor may recognize several different chemokines and vice versa (Table 1) (Mantovani, 1999). Among CXCR family members, CXCR3, which is predominantly expressed on immune cells, including activated T lymphocytes and natural killer cells, and selectively binds to CXCL9, CXCL10, and CXCL11 in a context depended manner plays a crucial role in immunological processes (Allen et al., 2007).

**TABLE 1:** Human CXC Receptors and their cognate ligands (Bateman et al., 2021).

CXC receptors	Identifier	CXC chemokines	Expression
CXCR1	P25024	CXCL6, CXCL7, CXCL8	Neutrophils, NK cells
CXCR2	P25025	CXCL1, CXCL2, CXCL3, CXCL5, CXCL6, CXCL7, CXCL8	Neutrophils, NK cells
CXCR3-A	P49682	CXCL9, CXCL10, CXCL11	Activated T cells, NK cells, Th1 cells
CXCR3-B	P49682	CXCL4, CXCL9, CXCL10, CXCL11	Activated T cells
CXCR4	P61073	CXCL12	T cells, B cells, Neutrophils, Monocytes, Macrophages
CXCR5	P32302	CXCL13	B cells
CXCR6	O00574	CXCL16	Activated T cells
CXCR7	P25106	CXCL11, CXCL12	Monocytes, basophils, B cells

In mammals, there are more than a dozen CXCL genes. In humans, a cluster of CXCL genes lies on chromosome 4, that code a large subset of CXC chemokines wherein genes coding for CXCL1-8 form centromeric subcluster, CXCL9-11 form a telomeric subcluster, and CXCL13, a unique ligand of CXCR5 involved in B cell homing, is found away from these subclusters (Colobran et al., 2007). CXCL1-8, except CXCL4, have a tri-peptide ELR motif (glutamic-leucine-arginine) that binds to CXCR1 and CXCR2 and are widely implicated in neutrophil chemotaxis and angiogenesis (Colobran et al., 2007). In contrast, CXCL4 is an anti-angiogenic ELR-negative chemokine and is known to bind to a splice variant of CXCR3 (Lasagni et al., 2003). Other ELR-negative chemokines, CXCL9 and CXCL10, bind to CXCR3 and play a significant role in the chemotaxis of activated T lymphocytes and NK cells. The genomic organization of the human chemokine superfamily has been highlighted in a review by Colobran et al. (Colobran et al., 2007). Murine CXC chemokines are clustered on chromosome 5 (Nomiyama et al., 2001). In contrast, in zebrafish, the genomic organization of CXC chemokines is clustered on various chromosomes; mini-clusters of CXC chemokines are present on chromosomes 1, 5, 13, 14, 22, and 24 (Nomiyama et al., 2008).

From an evolutionary perspective, in humans, CXCR3 has evolved as a single gene, expressed by a vast array of cells, including monocytes (Cella et al., 1999), dendritic cells (García-López et al., 2001), neutrophils (Ichikawa et al., 2013), endothelial cells (García-López et al., 2001), neurons, and astrocytes (Goldberg et al., 2001). The broader and overlapping expression pattern of the CXCR3 receptor and its ligands indicate that it is essential to have a clear idea about the tissue/disease-specific expression pattern of these genes and the signaling mechanism of the CXCR3-CXCL cascade to develop tissue/disease-specific robust therapeutics. This review provides a systematic background and highlights the current status of research in this growing field. Additionally, the receptor is highly conserved among vertebrates and thus has the potential to be studied in the experimentally more amenable model organism zebrafish, which has been highlighted in the review. A summary of the area we have covered; 1) discovery of CXCR3 and its ligands, 2) evolution of CXCR3, 3) structural and functional aspects of the receptor and its subsequent signaling mechanisms, 4) expression pattern of CXCR3 in development and diseases, and 5) its role in wound healing and a wide variety of neurological and cardiovascular disorders.

### Discovery of CXCR3 and Its Ligands

CXCR3 is a seven transmembrane G protein-coupled receptor belonging to the class A (rhodopsin-like) family (Stone et al., 2017). Genes encoding human and murine CXCR3 are present on chromosome X (Lu et al., 1999). While humans have alternative splice variants for CXCR3, i.e., CXCR3A and CXCR3B, no isoforms have been reported for mice yet. Zebrafish display three paralogues (*cxcr3.1*, *cxcr3.2*, and *cxcr3.3*) and all located in tandem on chromosome 16 (Torraca et al., 2015).

Human CXCR3, also known as CXCR3A, has three predominant functional ligands; CXCL9, CXCL10, and CXCL11, all of which are interferon-gamma (IFN-γ) inducible (Van Raemdonck et al., 2015). While these ligands have 40% similarity, they have highly variable spatiotemporal expression patterns, expressed by distinct cell types in response to specific cytokines or Toll-like receptor ligands. Moreover, their potency varies; CXCL11 has the highest affinity for CXCR3A and is the most efficient ligand out of the three, with a highly potent chemotactic response in cells expressing CXCR3A (Van Raemdonck et al., 2015).

The human CXCR3 gene encodes a 45.65 kDa (415 aa) protein. Partial cDNA of human CXCR3 was cloned from a CD4^+^ T lymphocyte cDNA library in 1996 (Loetscher et al., 1996). This cDNA had an open reading frame of 1,104 bp encoding a protein with 368 amino acids and a molecular weight of 40.6 kDa. CXCR3 carries seven putative transmembrane regions in the sequence, consistent with G protein-coupled receptors, three potential N-glycosylation sites, nine serines, and one threonine in the intracellular COOH-terminal, which could be potential phosphorylation sites for receptor kinases. In T cells, IL-2 can induce CXCR3 expression *in vitro* (Loetscher et al., 1996). Overexpression of CXCR3 in cultured cells resulted in increased cytosolic Ca2+ and cell migration in response to two of its ligands; Mig (CXCL9) and IP-10 (CXCL10) (Loetscher et al., 1996). Partial cDNA of murine CXCR3 was cloned in 1998 from an αβTCR^+^ CD4^−^ CD8^−^ T cell cDNA library (Soto et al., 1998). It had an open reading frame of 1,101 bp coding for a 367 amino acid long protein with a molecular weight of 41 kDa (Soto et al., 1998). CXCR3 is present on the X chromosome in murine and humans (Lu et al., 1999; Loetscher et al., 1998).

The first non-mammalian CXCR3-like gene has been discovered in grass carp (*Ctenopharyngodon idella*) (Chang et al., 2007). The cDNA consisted of 1,261 bp encoding 341 amino acids weighing 38.5 kDa, carrying seven transmembrane helices and four potential N-glycosylation sites. Transcripts of this gene were detected in blood cells and the central nervous system of healthy fish (Chang et al., 2007).

**FIGURE 1:**
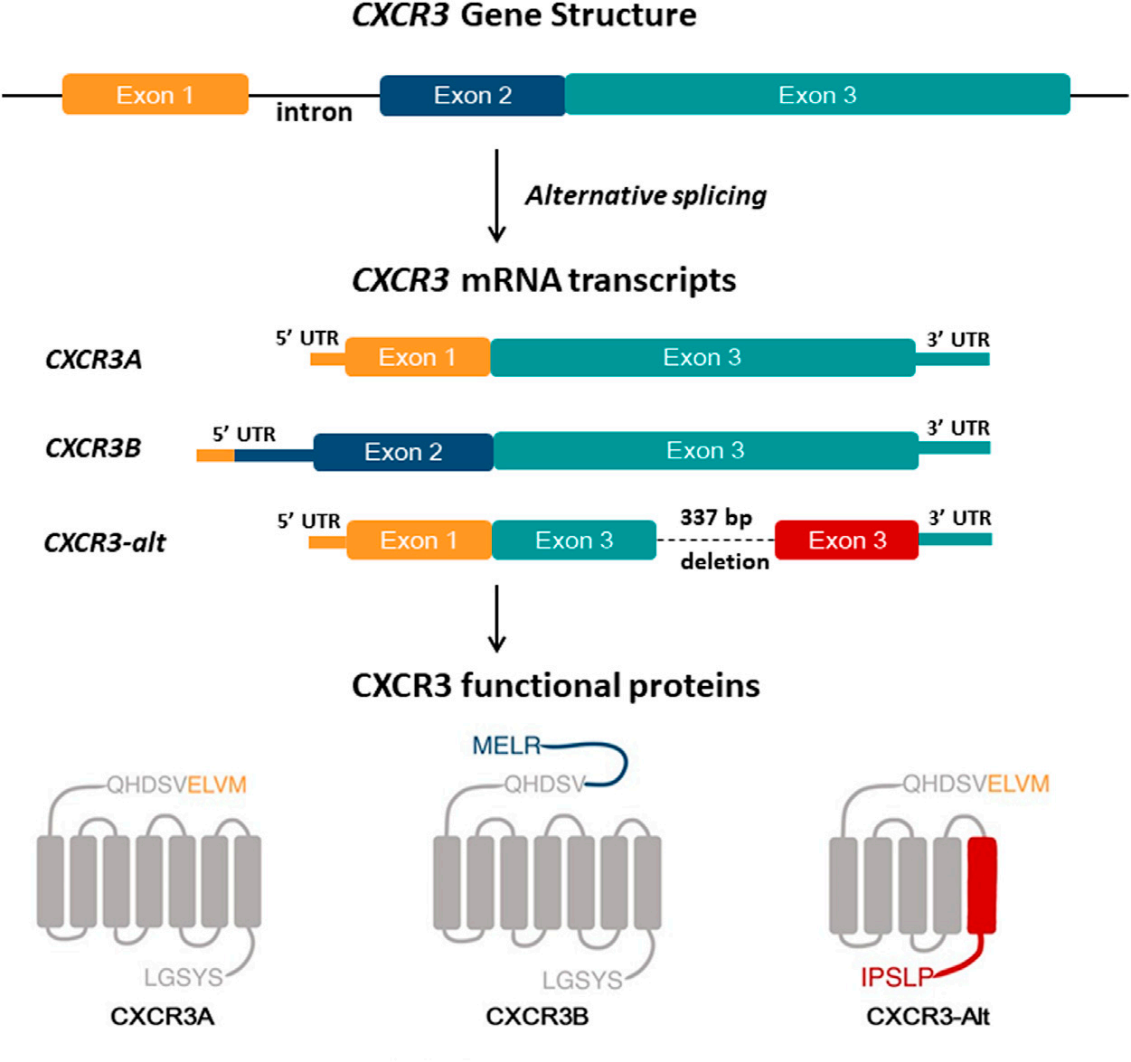
Overview of the human CXCR3 gene and CXCR3 isoforms structure. Human CXCR3 has three splice variants; CXCR3A is a spliced product of exon-1 and exon-3 of the CXCR3 gene. Alternative splicing of exon-2 and exon-3 results in CXCR3B, with a longer N terminal than CXCR3A by 52 extra amino acid residues. CXCR3-Alt is formed by deletion of 337 bp in exon 3, resulting in a protein having an N terminal and four transmembrane domains identical to CXCR3A and a different fifth transmembrane domain and C terminal than CXCR3A and CXCR3B.

In 2003, Lasagni et al. discovered a splice variant of human CXCR3 by using rapid amplification of cDNA ends (RACE) (Lasagni et al., 2003), subsequently named CXCR3B, while the original transcript was renamed CXCR3A, though it is still referred to as CXCR3. CXCR3B largely overlapped with the known CXCR3A but had a difference at the 5′ end due to alternative splicing (Figure 1) (Lasagni et al., 2003). CXCR3B has a longer extracellular NH2-terminal domain, with 52 amino acid residues. *In vitro* study on human microvascular endothelial cell line-1 (HMEC-1) showed that CXCL9, CXCL10, and CXCL11, bind with CXCR3A as well as CXCR3B. In contrast, CXCL4 binds specifically to CXCR3B but not to CXCR3A. Thus, a new ligand for CXCR3 was consequently discovered. CXCR3B transcripts were detected in the heart, skeletal muscle, liver, and kidneys (Lasagni et al., 2003). Subsequently, in 2004, Ehlert and his colleague discovered another variant of human CXCR3 generated by posttranscriptional exon skipping (Ehlert et al., 2004). The new variant CXCR3-alt is differed from CXCR3A due to its lack of bases 696–1,032. As a result of exon skipping, CXCR3-alt carries five transmembrane domains (Figure 1). CXCR3A expressing cells express a very low level of CXCR3-alt due to mRNA instability. However, despite the considerably lower surface expression levels, CXCR3-alt possesses some functional significance; chemotaxis in response to CXCL11 (Ehlert et al., 2004). CXCL4, a 101 amino acid long chemokine, also known as platelet factor-4 (PF4), was the first CXC chemokine to be discovered. It binds to heparin and is released during platelet aggregation (Deuel et al., 1977). CXCL4 is a functional ligand of CXCR3B and plays a crucial role in CXCR3B’s angiostatic activity (Lasagni et al., 2003).

Human CXCL10 was cloned in 1985 from U937, a human cell line treated with IFN- γ and named interferon-inducible protein 10-kDa (IP-10) (Luster et al., 1985). IFN- γ treatment led to the accumulation of IP-10 mRNA transcripts. IP10 showed significant homology with platelet factor-4 (PF-4) and β-thromboglobulin (β-TG), which are mediators of inflammation and wound healing *in vitro*, suggesting an inflammatory role for IP-10 (Luster et al., 1985). In response to IFN-γ treatment, IP-10 could be induced in many human cells, including monocytes, endothelial cells, keratinocytes, and fibroblasts (Luster and Ravetch, 1987).

CXCL9, also known as monokine induced by gamma interferon (MIG), was identified from a lymphokine-activated murine macrophage cDNA library (Farber, 1990). The murine gene was IFN-γ-inducible and was subsequently used to screen a human monocytic cell line (THP-1) treated with IFN-γ. The screen identified the human CXCL9 gene, which encodes a 103 amino acid long protein (Farber, 1993). The chromosomal location of human CXCL9 (MIG) and CXCL10 (IP-10) is in the q21.1 region on chromosome 4, wherein these genes form a mini-cluster in a head-to-tail orientation (Lee and Farber, 1996).

The chemokine CXCL11, also known as I-TAC (IFN-inducible T-cell alpha chemoattractant), binds CXCR3 with the highest affinity, first characterized in 1998 (Cole et al., 1998). The gene was isolated from a cDNA library of IFN-γ stimulated primary human astrocytes. I-TAC was mapped to chromosome 4 and showed high sequence similarity with IP-10 and MIG. Due to the similarity, it was predicted to have a similar modus operandi as its non-ELR family members in the context of a T cell response. As expected, it was discovered that I-TAC could induce a robust chemotactic response in activated T cells (Cole et al., 1998). It was also found that IP-10, MIG, and I-TAC could all interact with the CXCR3 receptor, though I-TAC was revealed to be the most potent and efficient CXCR3 ligand (Cole et al., 1998).

### Evolution of the CXCR3 Receptor

To unravel the evolutionary history of a chemokine receptor, it is imperative to study when chemokine receptor genes began to appear in chordate genomes. Chemokines are not present in cephalochordates, the earliest-branching subphylum of chordates (Bajoghli, 2013) as well as in tunicates (Huang et al., 2008; Nordström et al., 2008). Chemokine receptors first appeared in jawless vertebrates like the sea lamprey (*Petromyzon marinus*), and this repertoire of chemokine receptors continued to evolve in jawed vertebrates as well with the cartilaginous fish, elephant shark (*Callorhinchus milii*) having at least 13 putative chemokine receptors (Bajoghli, 2013). The diversity of receptors continued expanding with the emergence of bony fish, which researchers believe is an outcome of gene duplication events. This would explain why zebrafish (*Danio rerio*) and medaka (*Oryzias latipes*) have 32 and 28 chemokine receptor genes, respectively (Bajoghli, 2013).

**FIGURE 2:**
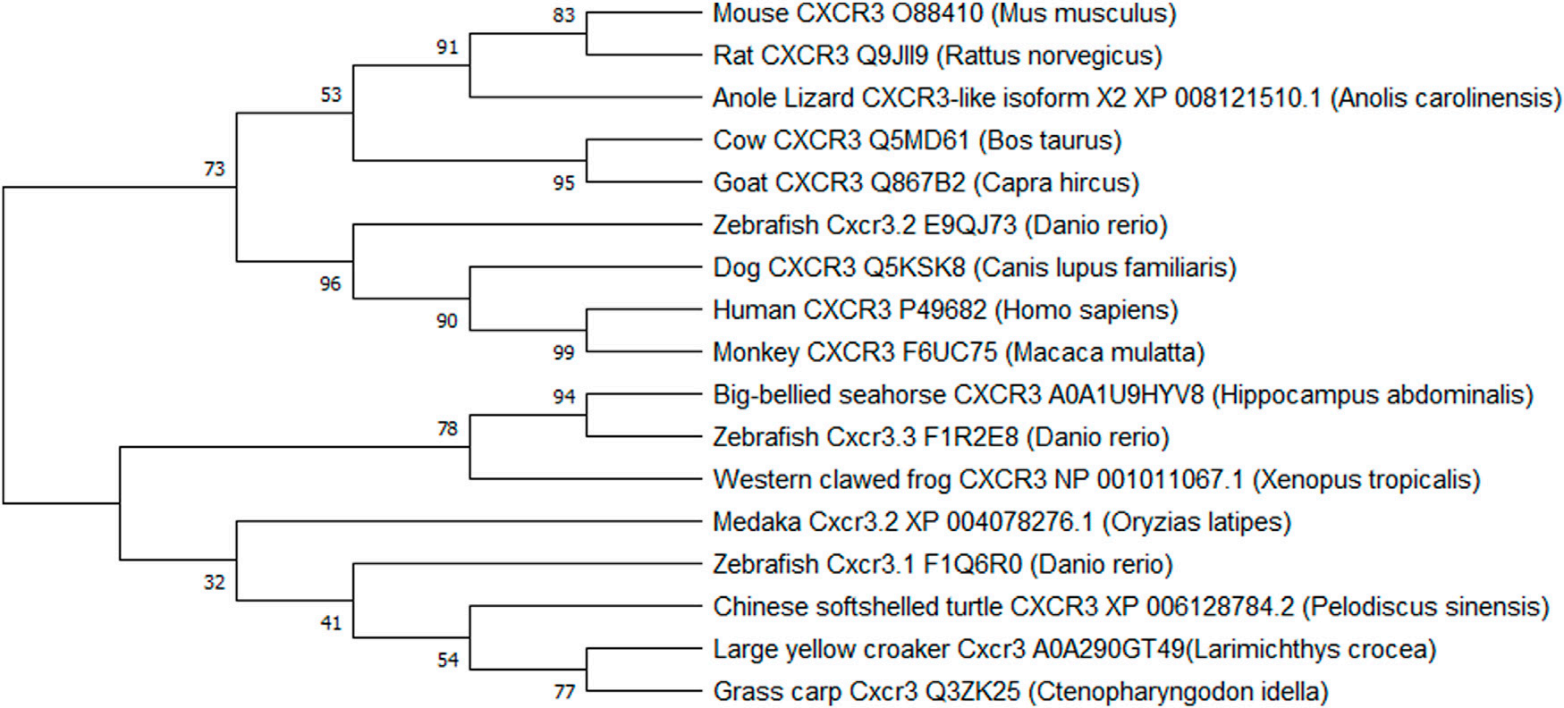
Phylogenetic tree representing the evolution of *CXCR3* and *CXCR3-like* genes in vertebrates. The evolutionary history was inferred by using the Maximum Likelihood method and Dayhoff matrix-based model (Dayhoff et al., 1978). Evolutionary studies were conducted in MEGA X (Kumar et al., 2018).

Historically it was believed that while chemokine receptor genes appeared quite early in vertebrate evolution, the CXCR3 receptor did not appear until bony fishes came into the picture. Later a CXCR3 homolog-like) was identified in a cartilaginous fish, the little skate (*Leucoraja erinacea*) (Zou et al., 2015). CXCR3 homologs were also identified in amphibians and reptiles, but surprisingly, not in birds (Nomiyama et al., 2011). Xu et al. (2014) have compared rainbow trout (*Oncorhynchus mykiss*) *Cxcr3a* and *Cxcr3b* genes with several other species, leading to quite a few conclusions. The fish Cxcr3a protein showed high amino acid identity to amphibian (frog) and reptile (turtle) Cxcr3a protein. The *Cxcr3a* and *Cxcr3b* genes showed conserved gene synteny in amphibians, lobe-finned fish, and ray-finned fish, but there was poor synteny between fish and mammals. Another observation revealed that some ray-finned fish had more *Cxcr3* paralogues due to local gene duplication events (Xu et al., 2014). These findings support the idea that *Cxcr3a* and *Cxcr3b* were present in the teleostomian ancestor and are thus, conserved in fish, amphibians, and reptiles. However, the *Cxcr3b* gene was possibly lost in mammals while both genes got lost in avian evolution (Xu et al., 2014). Figure 2 represents a phylogenetic tree for the evolution of the receptor in various vertebrates. It is apparent that zebrafish Cxcr3.2 is very closely related to human CXCR3 and validates its existence as the functional homolog. Since the receptor is evolutionarily conserved between zebrafish and humans, a wide variety of human diseases with CXCR3 involvement can be modeled and studied in zebrafish.

### Structural and Functional Aspects of CXCR3-Chemokine Interactions

**FIGURE 3:**
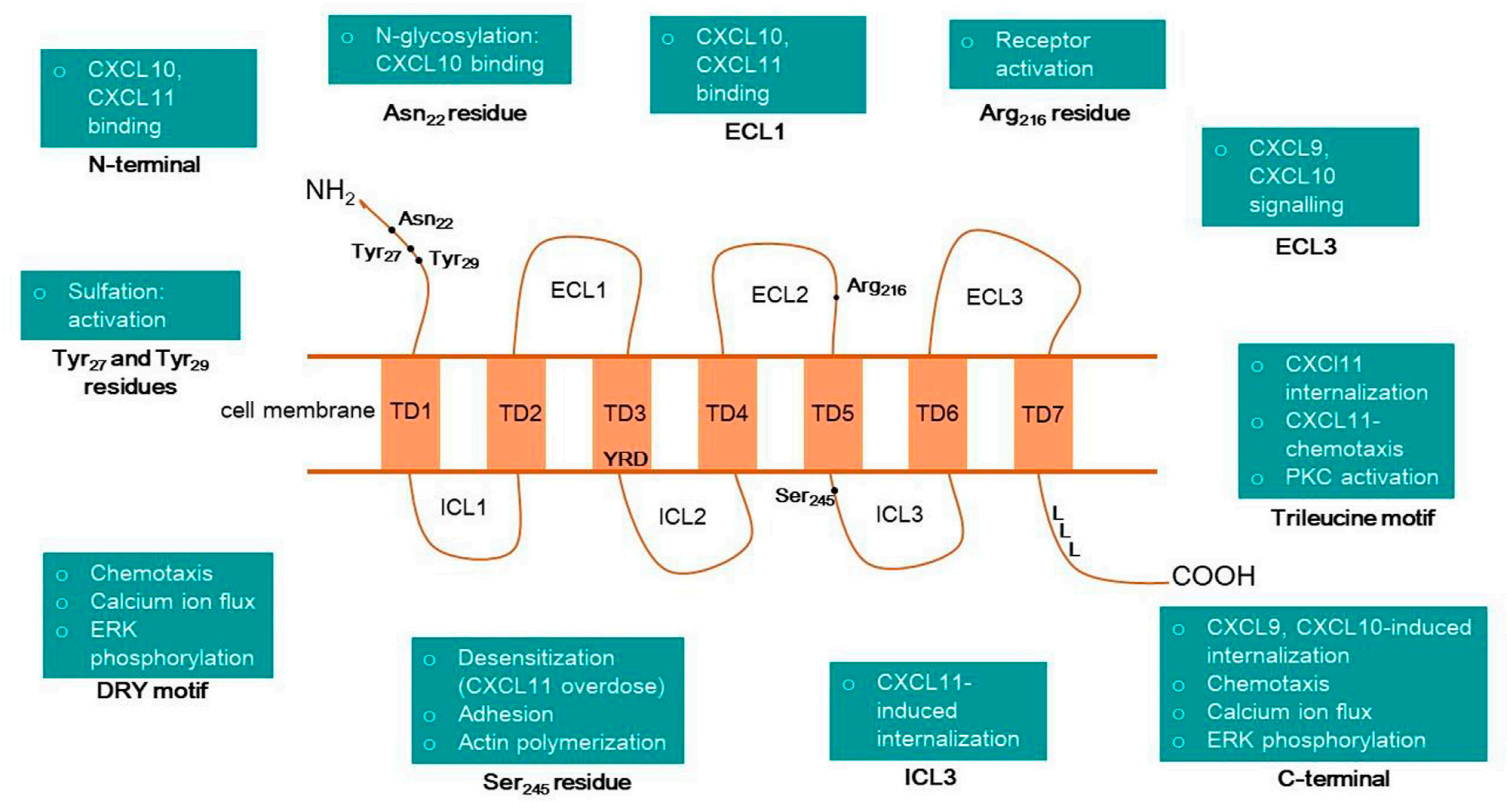
Overview of human CXCR3 structure and its motifs or amino acid residues associated with different cellular or molecular functions.

The CXCR3 protein is a seven-transmembrane, G protein-coupled receptor, with an N terminus extracellular domain, three extracellular loops (ECL1-3), three intracellular loops (ICL1-3), and a C-terminal intracellular domain (Figure 3). Xanthou and others have extensively studied the structure-function relationship between the extracellular domains of human CXCR3 and interacting chemokines using chimeric constructs of CXCR3 and CXCR1 with their domains interchanged. CXCR1 was chosen as it belongs to the same receptor subclass as CXCR3 and shares a 40% sequence identity. Additionally, it does not respond to CXCR3 ligands, which is vital for these chimeric studies (Xanthou et al., 2003). The study revealed that the N terminus and ECL1 play a pivotal role in binding CXCL10 and CXCL11, but ECL1 is not required for CXCL9 mediated signaling. Another important observation was that the lack of ECL2 in CXCR3 led to the complete erasure of signaling. These findings indicate that ECL2 possibly plays a role in receptor activation, with the conversion of inactive G to active G protein and that ECL2 is essential for all three ligands. It was also discovered that ECL3 is required for CXCL9 and CXCL10 mediated signaling, but not CXCL11 (Xanthou et al., 2003). The function of the intracellular and transmembrane domains of human CXCR3 was worked out by Colvin et al. Their study showed that chemotaxis, ERK phosphorylation, and calcium mobilization in response to all three ligands were mediated by the C-terminal cytoplasmic domain and a DRY sequence in the third transmembrane domain of CXCR3. CXCL9 and CXCL10-induced receptor internalization was facilitated by serine and threonine residues on the tail-end of the C-terminal along with the adaptor protein β arrestin-1, while CXCL11 required only ICL3 as part of a C-terminal-independent internalization process (Colvin et al., 2004).

Dagan-Berger et al. (2006) refuted the earlier belief, CXCL11 does not require the C-terminal of CXCR3 for its internalization and found that cell migration and receptor internalization in response to CXCL11 is regulated by a trileucine motif on the proximal end of the C-terminal.

CXCR3 receptor-ligand complexes get stabilized via aromatic and electrostatic interactions between the N-terminals of the three chemokines and CXCR3 (Raucci et al., 2014). CXCR3 shows the highest affinity to CXCL11, possibly due to salt bridges and hydrogen bonds formation (Raucci et al., 2014). Chemokines interact with CXCR3 in a 2-step model. The first step is recognition, wherein the receptor recognizes the “docking domain” of the ligand, the N loop. The second step is the binding, which depends on the position of the “triggering domain” or the flexible N-terminal of the chemokine (Clark-Lewis et al., 2003). CXCL10 has two hydrophobic clefts in the N loop and N-terminal, which interact with CXCR3 (Booth et al., 2002). Molecular docking studies confirmed these earlier findings, which solidified the hypotheses that N-loop and N-terminal of chemokines interact with CXCR3’s three ECLs and the N-terminal domain as well as the role of ECL1 in CXCL10 and CXCL11 signaling but not CXCL9 (Trotta et al., 2009). CXCR3 receptor’s N terminal ECL2 and ECL3 have negatively charged residues, while all ligands have positive residues in their N terminal (Trotta et al., 2009). Thus, electrostatic interactions occur between CXCR3 and its ligands. Additionally, phosphorylation sites are present in the binding domains of both the receptor and the ligands. Post-translational modifications of the CXCR3 have a crucial role in receptor-ligand interactions. A site-directed mutagenesis-based study identified the effects of sulfation on binding and activation of CXCR3 (Colvin et al., 2006). Sulfation of the Tyr_27_ and Tyr_29_ residues is required for CXCR3 activation, and the first 16 residues of the N-terminal are vital for maximal binding of CXCL10 and CXCL11. The Arg_216_ residue in ECL2 is crucial for activation but does not affect binding or internalization in response to the chemokines (Colvin et al., 2006). N-glycosylation of Asn_22_ and Asn_32_ residues is required for CXCL10 binding in fibroblast-like synoviocytes expressing CXCR3 (Sun et al., 2017). Figure 3 summarizes the role of various domains of the human CXCR3 receptor.

**FIGURE 4:**
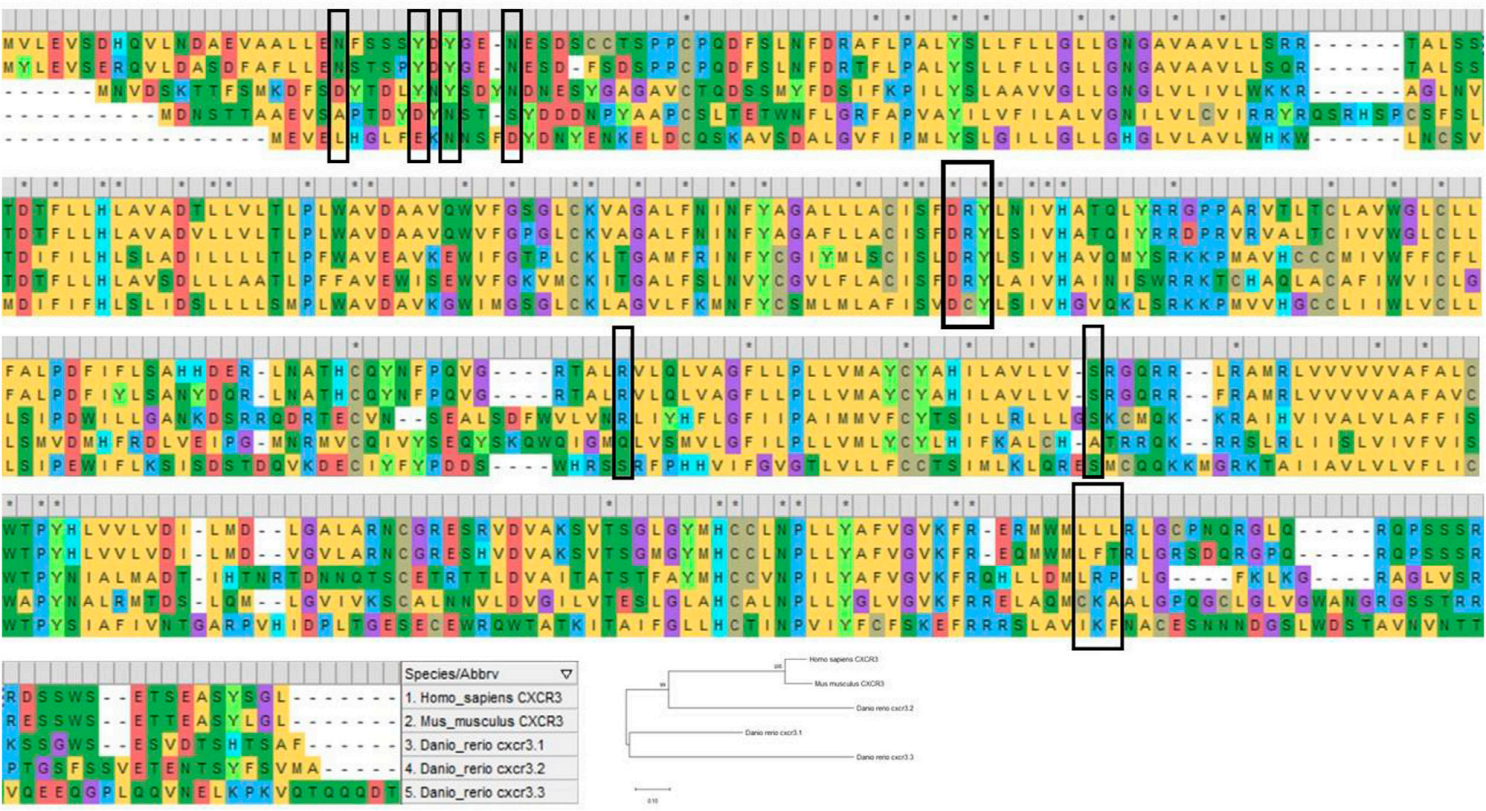
The image shows multiple sequence alignment of human CXCR3A, murine CXCR3 and zebrafish Cxcr3.1, Cxcr3.2, and Cxcr3.3. The highlighted residues (black box) show the conservation of essential domains in humans, mice, and zebrafish. The sequence alignment was done using MEGA X (Kumar et al., 2018).

While the human CXCR3 receptor’s domains have been extensively dissected for their structural and functional facets, very little has been revealed about the zebrafish counterparts. Unraveling the complexities of the receptor in a valuable model such as zebrafish, where immunological processes involving chemokines can be literally visualized in transparent live larvae, researchers can perhaps address more profound research questions pertaining to CXCR3 and its immunological implications in disease and regeneration. Zebrafish carry three paralogs of *CXCR3*; *Cxcr3.1*, *Cxcr3.2*, and *Cxcr3.3* (Torraca et al., 2015). Upon aligning the protein sequences of Cxcr3.1, Cxcr3.2, and Cxcr3.3, with its murine and human counterparts, one can predict various functional roles for the receptor. The important motifs and domains are relatively conserved in zebrafish to a certain degree (Figure 4). The DRY sequence, vital for receptor signaling, is highly conserved in human, murine, and zebrafish CXCR3, except for an amino acid substitution in the central arginine residue in zebrafish Cxcr3.3. However, a possible explanation for this discrepancy has been elucidated by Sommer et al.; Cxcr3.3 acts as an atypical chemokine receptor; the arginine substitution may have resulted in a scavenging role for the receptor rather than a loss-of-function phenotype (Sommer et al., 2020). The slightly conserved DRY motif in the Cxcr3.3 receptor and the overall homology between the receptors allow binding and sequestering the ligands without any downstream signaling, leading to impaired chemotaxis (Sommer et al., 2020). Moreover, alignment analysis shows that most of the essential residues of human CXCR3 are shared by Cxcr3.1 (Figure 4), and thus, the zebrafish Cxcr3.1 receptor may be functioning similarly. In conclusion, there is tremendous potential in studying the signaling mechanism of the receptor in zebrafish to uncover newer and deeper aspects of the human CXCR3.

### Expression of the CXCR3 Receptor

Chemokine gradients are vital for surveilling immune cells to hone in on a target tissue; in most cases, the destination is an injured or inflamed environment. It is thus, not surprising that CXCR3 is predominantly expressed on immune cells. Human CXCR3 is largely expressed on IL-2 activated T lymphocytes and NK cells (Loetscher et al., 1996). It has also been identified on B cells (Loetscher et al., 1998), CD4^+^ and CD8^+^ effector, and memory T lymphocytes (Loetscher et al., 1996). Naïve T cells can be induced to express CXCR3 in response to dendritic cell-mediated activation and via IL-2 exposure *in vitro*. Other immune cells, including Th17 cells (Lim et al., 2008), regulatory T cells (Treg) (Koch et al., 2009), and γδT cells (Poggi et al., 2004), express significant levels of CXCR3. Th1 subset of cells expresses higher levels of CXCR3 as compared to Th2 (Loetscher et al., 1996; Kim et al., 2001). While CXCR3 is expressed on these cells at a basal level, it gets upregulated in inflammatory conditions (García-López et al., 2001). CXCR3 receptor plays an instrumental role in T cell maintenance, along with a myriad of different processes such as Th1 cell-mediated inflammation leading to protection against intracellular pathogens, allowing specific cells to enter or restrict them during moments of crisis and amplification of an immune response. CXCR3, when expressed on regulatory T cells, acts as a feedback system to curb an unnecessarily amplified T cell-mediated immune response (Groom and Luster, 2011). CXCR3 expression has been detected on dendritic cells in lymphoid organs and infiltrated lymphocytes at the site of inflammation (García-López et al., 2001), suggesting a role for the receptor in regulating leukocyte migration to lymphoid organs as well as inflammatory sites. Immunohistochemical analyses revealed that CXCR3 is present on macrophages in immune system organs like lymph nodes, tonsils, and thymus (García-López et al., 2001). Early experiments showed that lymphocytes isolated from inflamed tissue in conditions like vaginitis and ulcerative colitis expressed high levels of CXCR3 (Qin et al., 1998). Accumulated B cells in the synovium of patients with rheumatoid arthritis also showed increased CXCR3 expression (Nanki et al., 2009). Thus, CXCR3 plays a crucial role in immune cell homing to an inflammatory environment.

Ichikawa et al. (2013) elucidated the role of the CXCL10-CXCR3 axis using lung injury mice models. Flow cytometry revealed an upregulated expression of CXCR3 in neutrophils present in the inflamed lung tissue but not on naïve, non-infiltrated neutrophils. CXCL10 treatment could enhance oxidative bursts and chemotaxis in neutrophils expressing CXCR3 in an autocrine loop at the inflammation site of the damaged lung. CXCR3 is, thus, a key player in intensifying inflammation at a damage site via an autocrine mechanism through its associated chemokines (Ichikawa et al., 2013). CXCR3 has also been found on eosinophils, upregulated by IL-2 activation and downregulated by exposure to IL-10. CXCL9 and CXCL10 could induce chemotaxis in eosinophils via the cAMP-dependent PKA pathway (Jinquan et al., 2000a). This suggests a role for CXCR3 in mediating eosinophil trafficking to sites of allergic inflammation.

Human CXCR3 expression is not limited to the immune system, however, as evidenced by several reports stating its presence on cultured neurons (Coughlan et al., 2000), astrocytes and Purkinje cells of the cerebellum (Goldberg et al., 2001), endothelial cells (García-López et al., 2001), smooth muscle cells (García-López et al., 2001), Kupffer cells in the liver (García-López et al., 2001) and several more as detailed in Table 2. *Post mortem* tissues of patients with CNS disorders, including gliosis in patients of HIV, multiple sclerosis, ischemic infarcts, and astrocytic neoplasms, showed increased CXCR3 expression on astrocytes (Goldberg et al., 2001). CXCR3 could thus play a role in the accumulation of reactive astrocytes at inflammation sites. Prominent expression was also found in Purkinje cells, similar in both normal and diseased tissue, signifying a housekeeping role in maintaining the cerebellum (Goldberg et al., 2001). The splice variant of CXCR3, i.e., CXCR3B, has been detected in the heart, kidney, liver, and skeletal muscles as well (Lasagni et al., 2003). Thus, it is clear that CXCR3 tissue distribution includes the central nervous system, heart, liver, and kidney and is not just limited to circulating immune cells in the blood.

**TABLE 2:** Various cell types express CXCR3. IHC, immunohistochemistry; ISH, *in situ* hybridization; FACS, fluorescence-activated cell sorting.

Type of cell	Organism	Detection method	Notes	References
CD4^+^ cells	human	Northern blot	constitutive expression	Loetscher et al. (1996)
CD8^+^ cells	human	Northern blot	constitutive expression	Loetscher et al. (1996)
NK cells	human	Northern blot	constitutive expression	(Loetscher et al., 1996), (Loetscher et al., 1998)
B cells	human	Northern blot	constitutive expression	Loetscher et al. (1998)
Th1 cells	human	FACS	constitutive expression	Kim et al. (2001)
T cells	human	IHC	upregulated expression in activated, infiltrated T cells in rheumatoid synovial fluid, inflamed tissues in vaginitis and ulcerative colitis	Qin et al. (1998)
plasmacytoid monocytes	human	FACS	constitutive expression	Cella et al. (1999)
peripheral blood B cells	human	FACS	B cells found in both normal and rheumatoid arthritis (RA) patients; CXCR3 may assist in migration to synovium in RA	Nanki et al. (2009)
tumor B cells	human	IHC, FACS	CXCR3 expressed in tumor cells of lymphocytic leukemia, small lymphocytic lymphoma, MALT lymphoma and hairy cell leukemia	Jones et al. (2000)
Th17 cells	human	FACS	expressed in Il-17 secreting cells in inflamed tissues	Lim et al. (2008)
Treg cells	human	FACS	upregulated expression so T_regs_ can accumulate at infection sites and control immune response	Koch et al. (2009)
dendritic cells	human	IHC	myeloid-derived CD11c+ cells in normal lymph organs and germinal lefts	García-López et al. (2001)
macrophages	human	IHC	constitutive expression in thymic macrophages and alveolar macrophages	García-López et al. (2001)
endothelial cells	human	IHC	Expression in large- and medium-sized EC (ie, post capillary venules), in EC of large- and medium-sized vessels of the thymus, lymph node, and tonsil	García-López et al. (2001)
Kuppfer cells	human	IHC	Expression in liver	García-López et al. (2001)
Vδ1 T cells	human	FACS	expressed in subset of γδT cells in peripheral blood of HIV-infected patients	Poggi et al. (2004)
neurons	human	IHC	constitutive expression	Coughlan et al. (2000)
astrocytes	human	IHC	expressed in glial cells of brain, spinal cord	Goldberg et al. (2001)
Purkinje cells	human	IHC, ISH	Constitutively expressed in cerebellum	Goldberg et al. (2001)
microglia	human	RT-PCR, ISH	Constitutive expression	Biber et al. (2002)
neutrophils	human	FACS	expressed in infiltrated neutrophils in inflamed lungs, synovial neutrophils	Ichikawa et al. (2013), Hartl et al. (2008)
eosinophils	human	IHC	expression of CXCR3 in presence of IL-2	Jinquan et al. (2000a)
microvascular dermal endothelial cells	human	IHC	constitutive expression	Salcedo et al. (2000)
CD34^+^ cells	human	FACS	upregulated expression in presence of GM-CSF	Jinquan et al. (2000b)
mast cells	human	IHC	expression in mast cells of airway smooth muscle in asthma patients	Brightling et al. (2005)
pericytes	human	FACS	expressed in hepatic stellate cells in the liver	Bonacchi et al. (2001)
mesangial cells	human	IHC	expressed in mesangial cells of patients with IgA nephropathy and glomerulonephritis	Romagnani et al. (1999)
keratinocytes	human	FACS, IHC	expressed in keratinocytes of epidermis, involved in wound healing	Kroeze et al. (2012)
macrophages	zebrafish	ISH	*cxcr3.2*, *cxcr3.3* constitutive expression	Zakrzewska et al. (2010), Sommer et al. (2020)
neutrophils	zebrafish	FACS	*cxcr3.2* constitutive expression	Torraca et al. (2015)
dendritic cells, macrophages	medaka	FACS, ISH, RT-PCR	*cxcr3a* constitutive expression	Aghaallaei et al. (2010)

However, despite the splice variants having overlapping tissue distribution for constitutive expression, CXCR3A expression is upregulated while CXCR3B is downregulated in prostate cancer tissue biopsies (Wu et al., 2012). Moreover, ectopic CXCR3B expression in prostate cancer cells can inhibit cell movement and invasion (Wu et al., 2012), suggesting that CXCR3B may activate a tumor-suppressive signal. Perhaps, the evolution of two variants of the CXCR3 receptor was a way to fine-tune some diverse downstream physiological responses.

Expression of CXCR3 receptor in teleosts has been reported to be limited to innate immune cells so far. Gene duplication events in teleosts have led to the occurrence of redundant genes that may result in subfunctionalization. A paradigm of this was reported by Lu et al. (2017) wherein two paralogues *cxcr3.1* and *cxcr3.2*, found to be expressed in macrophages of ayu (*Plecoglossus altivelis*), grass carp (*Ctenopharyngodon idella*), and spotted green pufferfish (*Tetraodon nigroviridis*), had differential roles in macrophage polarization to M1 and M2, respectively. Macrophages are vital for pathogen clearance in inflammatory states as well as in tissue homeostasis. The polarization between pro-inflammatory M1 and anti-inflammatory M2 is crucial for a regulated and efficient immune response in response to specific environmental cues. Thus, the cost of having redundant genes is overcome by the benefit of having two paralogous genes regulating macrophage function. The role of Cxcr3.2 in macrophage chemotaxis to bacterial infection sites was investigated by using *cxcr3.2* mutant zebrafish (Torraca et al., 2015). Earlier, it was established using FACS that Cxcr3.2 expression occurs at 1day post fertilization (dpf) in phagocytes expressing the macrophage-specific marker *csf1r*, but not in cells with the neutrophil marker *mpx* (Zakrzewska et al., 2010). However, it was later revealed that FACS-sorted macrophages maintain Cxcr3.2 expression levels at 6dpf and are expressed on neutrophils as well (Torraca et al., 2015). Another teleost, medaka (*Oryzias latipes*), have three paralogues; *cxcr3a*, *cxcr3b,* and *cxcr3c*. Transgenic *cxcr3a:gfp* reporter medaka fish showed *cxcr3a* expression in innate immune cells as well as cells of the central nervous system and adult fish skin (Aghaallaei et al., 2010). Even though the expression of this receptor by mononuclear phagocytes seems to be conserved between teleosts and mammals, it is unknown whether any of the zebrafish *cxcr3* paralogs play a role in T cell recruitment to inflammatory environments like their mammalian counterparts.

It is apparent from the expression pattern of the receptor that CXCR3 is involved in a vital process like cell migration, a cornerstone of the immune system, and may be involved in the pathophysiology of several diseases of the nervous system, circulatory system as well as autoimmune disorders.

### Signaling Through CXCR3

Like a typical chemokine receptor, CXCR3 also transduces signals in response to the binding of its cognate ligands CXCL9, or CXCL10, or CXCL11, via activation of G proteins and subsequent downstream effectors. Deducing the molecular events triggered by a ligand activating the receptor can have vast implications for therapeutic strategies aimed at controlling diseases involving CXCR3. Various reports over the decades have significantly contributed to unraveling the signaling pathway for this cell surface receptor. While CXCL9 and CXCL10 bind to CXCR3 competitively, CXCL11, the most potent ligand of CXCR3, binds allotropically to the active conformation of the receptor (R*) and can bind with a very high affinity to the uncoupled form as well (Cox et al., 2001). Consequently, due to different binding sites in the receptor, CXCL11 behaves as a highly effective agonist.

CD34^+^ progenitors stimulated with Granulocyte-macrophage colony-stimulating factor (GM-CSF) express CXCR3 and undergo chemotaxis and adhesion via phosphorylation of Syk protein kinase upon activation by CXCL9 and CXCL10 (Jinquan et al., 2000b). Experiments performed by Bonacchi et al. (2001) led to fascinating insights on potential signaling mechanisms for CXCR3. Tissue-specific pericytes (hepatic stellate and glomerular mesangial cells) expressing CXCR3 are crucial in tissue repair. In hepatic stellate cells, activation of CXCR3 by CXCL10 led to chemotaxis and proliferation via the Ras/ERK cascade along with Src phosphorylation. This is accompanied by increased phosphatidylinositol 3-kinase (PI3K) activity. In mesangial cells, agonist activation of CXCR3 led to increased cell division via stimulating ERK (Bonacchi et al., 2001). These experiments revealed different biological responses via CXCR3 signaling pathways, especially in the context of tissue repair and wound healing, since proliferation and migration of vascular pericytes are crucial for these processes. In contrast, it has been reported that COS-7 cells heterologously expressing CXCR3 in culture activates the p44/p42 MAP kinase and Akt/PI3K pathways in response to CXCL11, and these pathways are also active in CXCR3-expressing T cells (Smit et al., 2003). However, chemotaxis could not be prevented by treatment with specific inhibitors aimed at abolishing these pathways, in turn solidifying their assertion that the pathways in question are not required for CXCR3-mediated cell migration. Chemotaxis in CXCR3 expressing cells occurs via phospholipase C pathways and phosphatidyl kinases other than class I PI3Kγ. Moreover, CXCL11-mediated chemotaxis could be blocked with pertussis toxin (PTX) (Smit et al., 2003), which implied CXCR3 coupled with Gα_i_. Conversely, in intestinal myofibroblasts, downstream biological responses and actin reorganization in response to CXCR3 activation by its ligands could not be blocked by the pertussis toxin (Kouroumalis et al., 2005), indicating that CXCR3 follows a mechanism void of Gα_i_ coupling. This difference is due to two splice variants of CXCR3 (Kouroumalis et al., 2005). The receptor believed to show coupling with Gα_i_ is CXCR3A, while the contradictory phenomenon mentioned above was observed for CXCR3B, the alternatively spliced variant of CXCR3.

Berchiche and Sakmar (2016) delved deeper into the differing signaling routes followed by different splice variant receptors. While CXCR3A couples to Gα_i/o_ proteins, is PTX-sensitive, can activate MAPK pathways, and recruit β-arrestins along with several other processes mentioned above, it does so with varying effectiveness for varying agonists. CXCR3B shows significantly lowered Gα_i_ activity and only responds to CXCL11 (Berchiche and Sakmar, 2016). Certain ligands could induce β-arrestins recruitment in a Gα_i_ independent manner (Berchiche and Sakmar, 2016). Quantitatively distinct cellular processes invariants, in response to differing agonists, with some receptor-ligand pairs appearing more effective than others, is due to biased agonism.

In CXCR3-alt, there is a very weak ERK1/2 phosphorylation and no Gα_i_ activation (Berchiche and Sakmar, 2016). Differential signaling mechanisms by the variants could be mainly attributed to their structures. CXCR3A has Tyr27 and Tyr29 residues that are sulfated and essential for chemokine binding. CXCR3B has a longer N terminus with two more tyrosine residues that could act as potential sulfation sites. These additional sulfation sites might be sulfated or influence the sulfation of the tyrosine residues shared by the two isoforms, resulting in weakened Gα_i_ activation by CXCR3B. The truncated CXCR3-alt receptor lacks the phosphorylation sites essential for β-arrestin recruitment (Berchiche and Sakmar, 2016). These different structures allow the fine-tuning of cellular responses upon binding a specific chemokine.

It was later revealed that CXCL9, CXCL10, and CXCL11, along with CXCL4 (a reported ligand for CXCR3B) (Lasagni et al., 2003), could activate Gα_i_ at CXCR3A but not at CXCR3B. Recruitment of β-arrestin was also comparatively lower for CXCR3B as compared to CXCR3A. Only CXCL11 could recruit β-arrestin to CXCR3B, and the interaction is restricted to the plasma membrane. CXCR3A-β-arrestin interaction is endocytic (Smith et al., 2017). Despite being weaker, CXCR3B is a β-arrestin biased receptor, possibly due to differential GRK recruitment due to conformation or residue changes. These findings buttress the thought that alternative splicing and biased agonism are processes required for precise and fine-tuned chemokine signaling. With this ideology, it would be wrong to call chemokine-receptor pairs redundant.

### Role of CXCR3 in Wound Healing

The regenerative wound healing process is marked by overlapping phases; hemostasis, inflammatory, proliferative, and remodeling. A precise understanding of the wound healing process and identifying the exact point at which CXCR3 plays a role in tissue regeneration will develop strategies to intervene and treat debilitating chronic wounds. Following hemostasis, there is an immigration of innate immune cells to the wound site; neutrophils act as the first responders and begin to clear debris from the damaged site. Eventually, monocytes and macrophages engulf pathogens, debris, and dead neutrophils (LeBert and Huttenlocher, 2014).

Like other vertebrates, neutrophils arrive first in zebrafish and scavenge debris, followed by macrophages in the resolution phase (Li et al., 2012). Macrophages and neutrophils express the Cxcr3.2 receptor and rush to infection sites in response to its functional ligands, CXCL11-like chemokines (Torraca et al., 2015). It would not be far-fetched to imagine that immigration of these immune cells to the wound site would also be controlled via the CXCR3 receptor-ligand axes. Sommer et al. (2020) confirmed this, with tail amputation assays in zebrafish larvae showing reduced macrophage recruitment in *cxcr3.2* deficient larvae. Thus, zebrafish have a high potential to act as a disease model to understand the CXCR3 mediated regulation on cell recruitment in the different phases of wound healing owing to its tremendous regenerative capacity (Beffagna, 2019).

Similarly, in an apical periodontitis mouse model, CXCL9, the CXCR3 ligand is expressed in macrophages in the periapical tissue (Hasegawa et al., 2021). Application of a CXCR3 antagonist inhibited inflammatory cell migration leading to reduced lesion size of the apical periodontitis (Hasegawa et al., 2021), clearly implying a crucial role for the CXCL9-CXCR3 cascade in early inflammation.

The inflammatory phase is followed by a proliferative phase, marked by infiltration of fibroblasts which secrete extracellular matrix proteins including collagen, fibronectin, and proteoglycans, along with neovascularization (Rees et al., 2015). CXCR3 seems to be involved in the phases following these events, i.e., the late phase of wound healing involving remodeling and reepithelization. In a full-thickness excision wound model, *Cxcr3* null mice showed delayed wound closure and a plethora of impaired wound healing phenotypes (Yates et al., 2007). After 90 days, while the wild-type mice showed functional regeneration, the *Cxcr3* null mice showed the wound in a hypercellular state with a haphazardly arranged matrix, indicating poor remodeling of collagen (Yates et al., 2007). Moreover, lack of CXCR3 resulted in a thick keratinized scar with disorganized dermis underlying it, similar to hypertrophic scars in humans (Yates et al., 2010). It was evident from these findings that CXCR3 acts precisely in between the transition of a wound from a highly active cellular and inflammatory environment to a matured matrix and a remodeled epithelium. The remodeling phase in the normal wound healing process is marked by a distinct lack of cells; they are replaced by neatly arranged collagen fibrils; this phenotype was lost in the CXCR3 null mice (Yates et al., 2012). The hypertrophic scar formation in CXCR3 null mice suggests a role for CXCR3 in the reepithelization process in wound repair. Keratinocyte migration is vital for proper reepithelization, is controlled by CXCR3 signaling via Calpain (Yates et al., 2012). CXCL11, expressed by dedifferentiating keratinocytes at the wound’s edges, promotes motility in CXCR3-expressing basal keratinocytes by reducing their adhesion (Satish et al., 2005). Interestingly wild-type fibroblast transplantation into the wounds of CXCR3 null mice led to an organized collagen matrix with increased tensile strength (Yates et al., 2012), suggesting CXCR3 expressing fibroblasts play a crucial role in organized collagen matrix development leading to wound healing.

Another important hallmark of the transition from a proliferative state to a differentiated state of a wound is the reduction in angiogenesis, which occurs just before the onset of the remodeling phase. CXCR3 mediated signaling is vital for suppressing uncontrolled angiogenesis (Huen and Wells, 2012). Keratinocytes expressing CXCL10 and CXCR4 in the resolving phase cause the dissolution of newly formed blood vessels, which persist in the wounds of CXCR3 null mice (Huen and Wells, 2012), indicating CXCR3 is crucial for blood vessel dissolution in wound healing. In primary human microvasculature endothelial cell culture, only the cell population in the S/G2-M phase express CXCR3, and their proliferation could be blocked by the exogenous supply of CXCR3 ligands, implying that CXCR3 had a definite angiostatic effect (Romagnani et al., 2001). It was later shown that the angiostatic activity of the ligands (including CXCL4)is facilitated by the splice variant CXCR3B (Lasagni et al., 2003). In the HMEC-1 endothelial cell line, overexpression of CXCR3A led to survival while CXCR3B led to apoptosis in response to CXCR3 ligands (Lasagni et al., 2003).

CXCL10 inhibits VEGF (vascular endothelial growth factor) induced endothelial cell motility and tube formation via CXCR3 activation, which occurs through a PKA-mediated inhibition of Calpain (Bodnar et al., 2006). Pericytes provide mechanical stability to microvessels and are vital for vessel maturation, vascular remodeling, regression, and maintenance (Bodnar et al., 2013). Pericytes treated with IFN-γ could promote *in vitro* vessel dissociation and inhibit microvascular endothelial cord formation by activating CXCR3 on endothelial cells (Bodnar et al., 2013), suggesting the role of CXCR3 in the pruning of overproduced blood vessels during the wound repair process.

A recent study showed a correlation between CXCR3 signaling and impaired wound healing in diabetic wounds (Okonkwo et al., 2020). The pruning of microvessels is weakened in diabetic wounds, translating into a delay in the maturation of the capillaries. Moreover, pericyte recruitment is negatively impacted in diabetic wounds. This study also showed decreased CXCR3 and CXCL10 in diabetic mice, prompting a link between CXCR3 signaling and wound resolution.

In conclusion, efficient wound healing requires a significant contribution of CXCR3 signaling, corroborated by the severely compromised wounds in mice lacking the receptor.

### Role of CXCR3 in Diseases

CXCR3 plays a pivotal role in mobilizing cells in response to a chemical signal, often associated with pathological conditions, wherein immunocompetent cells are drawn to infection sites. Several diseases occur due to infiltration of immune cells at the wrong place, wrong time, or an inappropriate amount. CXCR3 has been widely implicated in various diseases like diabetes (Fallahi et al., 2016), vitiligo (Kuo et al., 2018), tumor progression, and cancer (Reynders et al., 2019), which have been extensively reviewed elsewhere. Judging from the expression pattern of the receptor, as detailed in Table 2, it is not surprising to find vital roles for CXCR3 in diseases of many organs, including the brain (Szczuciński and Losy, 2007), heart (Szentes et al., 2018), skin (Kuo et al., 2018), liver (Larrubia et al., 2008), lungs (Henrot et al., 2019), and kidney (Enghard et al., 2009). Here our focus will be on the aspect of CXCR3 in neurological and cardiovascular diseases.

### Role of CXCR3 in Neurological Diseases

The central nervous system (CNS) is considered an “immunologically-privileged” site due to unique advantages conferred to it by the blood-brain barrier, which separates the CNS from the immunological minefield outside it. However, diseases such as multiple sclerosis (MS), viral encephalitis, etc., rely on immune cell recruitment and infiltration to the CNS for pathogenesis (Jinquan et al., 2000b). Chemokines play a key role in neuromodulation and neuroinflammation. In the CNS, cells including microglia, astrocytes, neurons, oligodendrocytes, etc., relay chemokine signals and respond to them through chemokine receptors. In a diseased condition, neuronal and glial cell injury mediated by microglia occurs primarily due to pro-inflammatory cytokines, eventually leading to trans-endothelial migration of immune cells across the blood-brain barrier and subsequent neuronal damage (Brightling et al., 2005).

A study by Goldberg et al. (2001) about the expression of CXCR3 on cells of the CNS in diseased conditions led to some fascinating insights into the receptor’s involvement in a wide variety of maladies. For instance, in HIV-positive patients, CXCR3-positive astrocytes were found surrounding cerebral infarcts, and these cells decreased in numbers with distance from the infarcted tissue. A similar phenomenon could be seen in patients who had suffered from an ischemic stroke, where an accumulation of CXCR3-positive astrocytes could be seen around infarcts (Goldberg et al., 2001). Reactive astrocytes predominantly expressing CXCR3 may indicate their role in reactive gliosis development in several infectious or inflammatory processes in the CNS. CXCR3 involvement has also been implicated in disorders such as Human T-lymphotropic virus type 1-associated myelopathy (Ando et al., 2013), chronic pain, hyperalgesia (Li et al., 2020), and neuroborreliosis (Židovec Lepej et al., 2005). Following are some selected major disorders of the CNS known to have CXCR3 involvement.

#### Multiple Sclerosis

Multiple sclerosis (MS) is considered an autoimmune disease where the body’s immune system attacks myelinated axons in the CNS and subsequent destruction of axons and myelin. It occurs due to the infiltration of T cells into the brain. Increased CXCL9 and CXCL10 expression and presence of CXCR3+ T cells in the cerebrospinal fluid (CSF) of the MS patients indicate the implication of CXCR3 in the MS lesion formation. (Sørensen et al., 1999). Later it was identified that Human brain microvascular endothelial cells and astrocytes produce IFN-γ inducible chemokines in developing lesions which acts as homing signals for CXCR3-expressing T lymphocytes (Salmaggi et al., 2002). Therefore, MS lesion development is a three-step process; first, activated T cells in the perivascular space express IFN-γ, then the surrounding IFN-γ exposed glial cells express CXCL9 and CXCL10, leading to CXCR3-expressing T cells infiltrating the inflammation site. MS patients, after clinical relapse, showed decreased CXCL10 but an increased number of CXCR3-positive T cells (Mahad et al., 2002), (Balashov et al., 1999). Thus, MS lesion progression occurs due to the CXCR3-positive T cells infiltration into the CNS, and therapeutic targeting or blocking of the receptor could have tremendous potential for MS treatment.

#### Alzheimer’s Disease

Alzheimer’s disease is manifested as amyloid β (Aβ) depositions and the formation of neurofibrillary tangles in the brain, which leads to irreparable neuronal damage (Goedert et al., 1991). CXCR3 is expressed constitutively on neurons in various tissues of the human CNS, including the neocortex, cerebellum, spinal cord, and striatum (Xia et al., 2000). In Alzheimer’s patients, while CXCR3 expression level remained unaltered, its ligand CXCL10 was found to be upregulated in astrocytes, which remained associated with senile plaques, characteristic of Alzheimer’s disease (Xia et al., 2000). Cerebrospinal fluid of patients with mild Alzheimer’s disease shows higher levels of CXCL10 than healthy individuals (Galimberti et al., 2003), which indicates a role of this molecule at the onset of the disease when there is prominent inflammation. These findings suggest a potential link between the CXCL10-CXCR3 axis in cell-cell communication between neurons, astrocytes, and microglia, which could play a role in neuronal injury.

Calcium ion dysregulation has been shown in fetal neuronal cultures in response to CXCL10 exposure (Sui et al., 2006). Apoptosis of neurons and other cells in the brain is a hallmark of Alzheimer’s disease. The binding of the ligand CXCL10 with CXCR3 leads to mitochondrial damage and subsequent release of cytochrome c, which kick-starts the intrinsic apoptosis cascade. (Sui et al., 2006).

It has been indicated that microglia in the CNS can modulate the pathological course of the disease (Krauthausen et al., 2015). While they generate reactive oxygen species, pro inflammatory cytokines and other neurotoxins, they also release enzymes capable of degrading Aβ plaques and mediate their phagocytosis. In a CXCR3-deficient mouse model, there was diminished Aβ concentration in the brain and reduced plaque formation, along with reduced behavioral impairment, indicating that morphological changes in the brain have functional implications. It was hypothesized that CXCR3-deficient microglia could phagocytose Aβ more efficiently, and an incomplete degradation of Aβ by these microglia could induce plaque formation (Krauthausen et al., 2015). CXCR3 plays a role in this process, possibly by blocking the mobilization of the microglia leading to reduced plaque formation.

An *in vitro* assay showed that CXCR3-deficiency in microglia increases amyloid β phagocytosis, and inhibition of CXCR3 augments microglial phagocytosis of the plaques (Krauthausen et al., 2015). In agreement, CXCL9 and CXCL10 supplementation led to a decreased uptake of amyloid β (Krauthausen et al., 2015). These findings suggest that CXCR3 plays a pivotal role in the pathology of Alzheimer’s disease and could be a potential therapeutic target.

#### Dengue Viral Disease

Dengue virus, a mosquito-borne pathogen, can cause hemorrhagic fever and shock. The pathogenesis in dengue involves neuroinflammation and neurodegeneration by evoking inflammatory cytokines and chemokines (Niranjan et al., 2019). Viral infections can rupture the blood-brain barrier and allow immune cells to cross over to the brain, leading to a wide range of immunopathological processes (Hsieh et al., 2006). Interestingly, in this condition, the role of CXCR3 seems to be protective rather than pathogenic. CXCR3 aids in the recruitment of CD8^+^ T cells to the site of infection, a widespread defense mechanism of the host against viruses. Similarly, *Cxcr3* ^*−/−*^ mice showed diminished viral elimination and paralysis (Hsieh et al., 2006). Therefore, for a therapeutic intervention, increasing CXCR3 expression on T cells may encourage complete purging of the virus from the system.

#### Glioblastoma

Glioblastoma, the most aggressive form of glioma, is a debilitating human cancer of the brain. The role of CXCR3 in this disease has been investigated to evaluate its potential as a biomarker or drug target. Pharmacological inhibition of Cxcr3 in murine malignant glioma (GL261) model showed antitumor progression effect (Liu et al., 2011), suggesting the connection between CXCR3 and glioma progression and CXCR3 could be a potential therapeutic target for glioma. Evidence suggests CXCR3 works as an oncogene by inducing glioblastoma multiforme (GBM) cell invasion and migration, and high CXCR3 expression correlates with poor survival in primary GBM patients (Pu et al., 2015). Taken together, CXCR3 has the potential as a biomarker and pharmacological target for preventing the progression of an aggressive brain tumor.

### Role of CXCR3 in Cardiovascular Diseases

Heart diseases are the leading cause of death worldwide, and research to look into possible treatments is the need of the hour. Cardiac dysfunction has been linked with heightened chemokine levels in the circulation (Tarzami, 2011). Most research has been aimed at unraveling the mechanisms of chemokines recruiting leukocytes to sites of inflammation and damage.

CXCR3 is expressed on vascular smooth muscle cells and endothelial cells, albeit at lower levels than T cells in healthy conditions (García-López et al., 2001). Several reports implicate CXCR3 and its ligands in the etiologies of various cardiovascular diseases, including atherosclerosis, hypertension, heart failure, and myocardial infarction (Altara et al., 2016a). Patients suffering from Kawasaki disease (Ko et al., 2015), a condition that shows vasculitis of the coronary arteries and often results in coronary artery aneurysms or myocardial infarctions (Newburger et al., 2016), show an increased level of CXCL10 along with CXCR3-activated T cells in the plasma, suggesting CXCR3-CXCL10 as a potential therapeutic target to suppress progression of the cardiac diseases (Ko et al., 2015). In rats, Cxcl9 and Cxcl10 showed elevated expression in both remote and near infarcted regions of the heart and remained high up to 16 weeks after myocardial infarction (Altara et al., 2016b). Similarly, serum CXCL10 levels are higher in patients with heart failure. Thus, CXCR3 ligands have great potential as biomarkers for heart diseases, and it would be helpful to discern the pathways involved to target the CXCR3 signaling axes as a treatment modality. Following are some cardiovascular diseases with known CXCR3 involvement.

#### Atherosclerosis

Atherosclerosis is a chronic inflammatory disorder of the coronary arteries, marked by the formation of atherosclerotic lesions initiated and maintained by a plethora of immune cells and cytokines. In atherosclerotic lesions and atheroma-associated cells, endothelial cells and macrophages secrete CXCL9 and CXCL11, and smooth muscle cells secrete CXCL10, which help in the recruitment and retention of CXCR3-expressing activated T cells (Mach et al., 1999). Coronary artery disease patients show a higher number of CXCR3-expressing peripheral blood mononuclear cells compared to normal individuals (Oliveira et al., 2009). In atherosclerosis, activated T lymphocytes recruited to the wound site detect LDL as antigen and produce pro-inflammatory molecules including IFN-γ and tumor necrosis factor (TNF) which leads to the induction of CXCL9, CXCL10, and CXCL11 expression by the cells of the region, leading to selective homing of Th1 cells expressing CXCR3 (Szentes et al., 2018). The CXCR3 ligands attract monocytes, dendritic cells, and macrophages that accumulate oxidized LDLs. Eventually, all these cells aid in plaque formation (Altara et al., 2016a). However, it has been found that CXCR3 may aid in lesion development in early atherogenesis but plays a minor role in the advancement of disease (Veillard et al., 2005).

It is known that atherosclerosis can be exacerbated with increased immune cell recruitment. CXCL10 and CXCL11 are expressed in human atheromas throughout the plaque development stages, which leads to increased recruitment of CXCR3-expressing macrophages and monocytes (Zernecke and Weber, 2010). A CXCR3-antagonist (NBI 74330) interfered with macrophage recruitment into plaques and subsequent accumulation (Zernecke and Weber, 2010). Thus, likely, macrophage-induced damage is a result of CXCR3 signaling.

Since CXCR3 involvement is at the onset of this disease, detailed investigation into the receptor acting as a therapeutic target could have implications towards blocking the progression of atherosclerosis.

#### Chronic Chagas Cardiomyopathy

Chronic Chagas cardiomyopathy (CCC) occurs due to infection by *Trypanosoma cruzi*, a parasitic euglenoid, which aggravates to become a condition marked by an inflamed myocardium with persistent chronic inflammation and infiltrated immune effector cells (Altara et al., 2016a).

*In vitro* stimulation with antigens of *Trypanosoma cruzi* on CD4^+^ and CD8^+^ T cells show co-expression of CXCR3 and IFN-γ (Gomes et al., 2005)*.* CCC patients show elevated levels of CXCR3-positive mononuclear cells in the myocardium, along with increased levels of plasma CXCL9 and CXCL10 (Nogueira et al., 2012). Moreover, it has been shown that elevated *CXCL9* mRNA expression level is directly proportional to the severity of myocarditis (Nogueira et al., 2012). Thus, it can be speculated that the CXCL9 and CXCL10/CXCR3 axes are master regulators of myocardial inflammatory cell migration.

However, while CXCR3 is involved in the migration of effector cells to the heart, it does not seem to be involved in the progression of the disease (Nogueira et al., 2012). Patients with severe CCC show lower CXCR3^+^ peripheral T cells and elevated CCR5 expression instead (Roffe et al., 2019). While it has been shown that CXCL10 is higher in patients with CCC, and polymorphisms in CXCL9 and CXCL10 are responsible for their altered expression patterns (Nogueira et al., 2012), CXCR3 expression on CD8^+^ cells is inversely proportional to the intensity of myocarditis (Roffe et al., 2019). This contradiction could be a possible result of CXCR3 internalization and subsequently lowered expression in response to saturating concentrations of CXCL9 and CXCL10.

The role of CXCR3 has been implicated in CD8^+^ cells migrating to the heart at the onset of *Trypanosoma cruzi* infection (Pontes Ferreira et al., 2019). Using the prime-boost vaccination method with *T. cruzi* antigens, it has been observed that *T. cruzi* antigens can induce a robust CD8^+^ cell response to protect susceptible mice against the infection. CXCR3 was found to be a key player in the migration of these cells to the infection site, as evidenced by increased parasite load and subsequent death of mice treated with anti-CXCR3 (Pontes Ferreira et al., 2019). This finding is important to consider while designing vaccines against intracellular pathogens; CXCR3 could essentially be used to guide specific CD8^+^ cells to the infected heart to control parasites from replicating.

#### Hypertension

Hypertension amalgamates various processes, including left ventricular hypertrophy, systolic and diastolic dysfunction, vascular dystrophy, etc., often resulting in symptomatic heart failure. High blood pressure frequently induces the left ventricular wall to thicken to reduce wall stress. In the event of “transition to failure”, the left ventricle dilates, and the ejection fraction falls down, due to which the heart cannot provide enough oxygen to the body, even to the cardiac muscle itself. Left ventricular hypertrophy is marked by increased cardiomyocyte size, altered extracellular matrix deposition, and tremendous tissue remodeling accompanied by fibrosis (Drazner, 2011).

Serum CXCL10 levels have been found to be significantly higher in patients with high blood pressure (Antonelli et al., 2008) and those suffering from essential hypertension (Stumpf et al., 2011). An increased fraction of CD8^+^ T cells and elevated levels of CXCR3 chemokines in hypertensive patients’ circulation has been important to forge a link between T-cell-driven inflammation and hypertension (Youn et al., 2013). Transverse aortic constriction mouse models showed increased cardiac recruitment of activated CD4^+^ and CD8^+^ T cells, accompanied by raised levels of Cxcl10, in ventricular tissues (Laroumanie et al., 2014). Patients with left ventricular dysfunction show increased levels of the CXCR3 ligands; CXCL9, CXCL10, and CXCL11 (Altara et al., 2015), which highlights an important diagnostic role for these ligands.

While CXCL10 was established as a biomarker, its direct role in maladaptive cardiac remodeling in response to a pressure overload was investigated in a mouse model (Koren et al., 2017). Adult cardiomyocytes appeared larger in volume upon exposure to CXCL10. CXCL10 infusion into mice showed an increase in ventricle/body weight. A significant discovery revealed that blocking Cxcr3 with an antagonist, AMG487, considerably suppressed cardiac remodeling and CXCR3-null mice displayed reduced hypertrophy (Koren et al., 2017). As a result, the CXCR3-CXCL10 axis could be an excellent therapeutic target for lowering hypertensive risks and subsequent heart failure.

## Discussion and Future Directions

Chemokines play a pivotal role in controlling immune cell trafficking and recruiting other factors to ensure the smooth sailing of the immunity of a system. The human CXCR3 receptor selectively binds the three non-ELR chemokines: CXCL9, CXCL10, and CXCL11, and is predominantly expressed on activated T cells, NK cells, and other effector cells of the immune system. However, not limited to just the immune cells; it is also expressed on non-immune cells, including neurons, endothelial cells, and several more, as elucidated earlier in this review. CXCR3 is mostly associated with the pathophysiology of various diseases. The CXCR3- CXCL9/CXCL10 axis has been largely implicated in heart failure and CNS disorders. In atherosclerotic lesions, endothelial cells and macrophages secret CXCL9 and CXCL10, which lead to the accumulation of CXCR3-expressing T cells in the lesions. CXCR3 signaling in atherosclerosis has been found to exacerbate the damage due to the accumulation of many cells expressing CXCR3 leading to a state of heightened inflammation and damage. Similarly, in multiple sclerosis and Alzheimer’s disease, CXCR3-expressing T cells infiltrate the CNS, and binding of CXCL10 to CXCR3 expressing cells leads to apoptosis of neurons and subsequent neuronal damages. CXCR3 acts as an oncogene by inducing invasion and migration of cells leading to malignant glioblastoma.

While CXCR3 appears to worsen the situation in most cases, its physiological role cannot be undermined. Thus, pharmacologically targeting the receptor can have unforeseen consequences on the organism’s functioning.

Since CXCR3 and its three ligands are expressed in numerous tissue types and play many different roles in physiological and pathological conditions, it is crucial to closely examine the mechanisms underlying CXCR3 signaling to develop robust organ-specific therapies. Zebrafish is an experimentally more amenable model to study vertebrate physiology and pathophysiology due to its external fertilization, transparency, ease of loss-of-function allele generation, and physiological and genetic similarity to humans. Thus, zebrafish would be a valuable model to get more insight into the CXCR3-CXCL9/10/11 signaling in pathophysiological conditions of different diseases. Research in this direction will undeniably lead to a more comprehensive understanding of this chemokine receptor and ways by which we can perturb its axis to prevent disease progression without hampering the physiological process.
